# A TRIAL TO IMPROVE MEDICATION SAFETY IN OLDER ADULTS: RECRUITMENT CHALLENGES HAVE GENERALIZABILITY IMPLICATIONS

**DOI:** 10.1093/geroni/igz038.2611

**Published:** 2019-11-08

**Authors:** Kathryn Anzuoni, Terry Field, Kathleen Mazor, Yanhua Zhou, Timothy Konola, Alok Kapoor, Lawrence Garber, Jerry Gurwitz

**Affiliations:** 1 Meyers Primary Care Institute, a joint endeavor of University of Massachusetts Medical School, Reliant Medical Group, and Fallon Health, Worcester, Massachusetts, United States; 2 University of Massachusetts Medical School, Worcester, Massachusetts, United States; 3 Reliant Medical Group, Worcester, Massachusetts, United States

## Abstract

For older adults, the transition from hospital to home is a high-risk period for adverse drug events, functional decline, and hospital readmission. Randomized trials of interventions to improve this transition must recruit potential subjects immediately after hospital discharge, when people are recovering and tired. Within a randomized trial assessing the impact of a pharmacist home visit to provide medication assistance immediately post-discharge, we determined whether individuals who enrolled were comparable to those who were invited but did not enroll, and described reasons for not enrolling. Individuals ≥50 years of age discharged from the hospital and prescribed a high-risk medication were eligible. We attempted to recruit individuals by phone within 3 days of discharge, and recorded reasons for not enrolling. Of 3,606 eligible individuals reached, 3,147 (87%) declined, 361 (10%) were enrolled, and 98 (3%) were initially recruited but did not complete a consent form. Individuals ≥80 years of age (odds ratio 0.45, CI 0.25, 0.78) and those with an assigned visiting nurse (odds ratio 0.64, CI 0.48, 0.85) were least likely to enroll. Among those who provided a reason for declining (2,473) the most common reason given was the belief they did not need medication assistance (22%). An additional 332 (13%) declined because they were receiving visiting nurse services. Recruiting older adults recently discharged from the hospital is difficult and may under-enroll the oldest individuals, limiting the ability to generalize findings across older patient populations. Researchers planning RCTs among newly discharged older adults may need creative approaches to overcome resistance.

